# Neuromesenchymal hamartoma of small bowel - an extremely rare entity: a case report

**DOI:** 10.1186/1477-7819-7-92

**Published:** 2009-11-27

**Authors:** Ekaterini Theodosiou, Grigorios Voulalas, Nikolaos Salveridis, Konstantinos Pouggouras, Kosmas Manafis, Konstantinos Christodoulidis

**Affiliations:** 1 1st Surgical Department, General Hospital, Kavala, Greece; 2 Department of Pathology, General Hospital, Kavala, Greece

## Abstract

Neuromuscular and vascular hamartoma (NMVH) is a very rare stricturing condition of the small intestine, occurring focally and causing recurrent obstructive symptoms or occult chronic gastrointestinal bleeding. Salas et al. (Neuromesenchymal hamartoma of the small bowel. J Clin Gastroenterol. 1990, 12 (6): 705-9) proposed the term of "Neuromesenchymal hamartoma" for the cases of NMVH with participation of mesenchymal tissues.

We present the case of a 60-year-old male patient admitted twice in a month with abdominal pain. On the third admission with clinical signs of acute abdomen, an exploratory laparotomy was performed. The clinical and laboratory findings that occurred after the patient's evaluation, the intraoperative findings and the pathological features of this lesion are reported.

## Introduction

Neuromuscular and vascular hamartoma (NMVH) is a very rare stricturing condition of the small intestine, occurring focally and causing recurrent obstructive symptoms or occult chronic gastrointestinal bleeding. It is a hyperplasia of varied tissue of the submucosa such as smooth muscle bundles, peripherals nerve tracts, vessels and ganglia. In our case there is an additional pathological feature: the diffuse fatty infiltration of the submucosa. Salas et. al. proposed the term of "Neuromesenchymal hamartoma" for the cases of NMVH with participation of mesenchymal tissues [1].

## Case report

**Figure 1:**
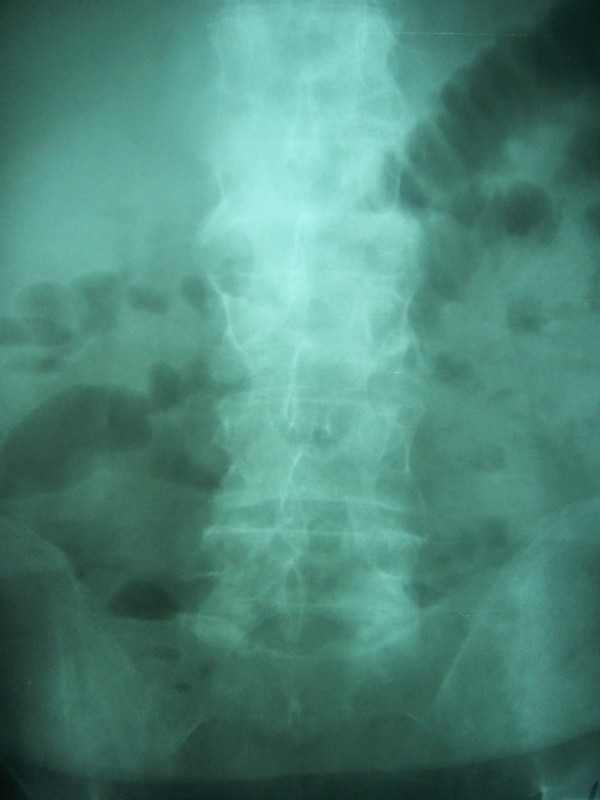
**On simple x-ray, signs of intestinal obstruction were found**.

A 60-year-old male patient was admitted twice in a month with abdominal pain and sings of intestinal obstruction on X-ray (figure 1). Both times the patient responded to conservative therapeutic measures and was discharged the 5^th ^day. The past medical history included hypertension and neither previous gastrointestinal disease nor surgical intervention was reported.

**Figure 2:**
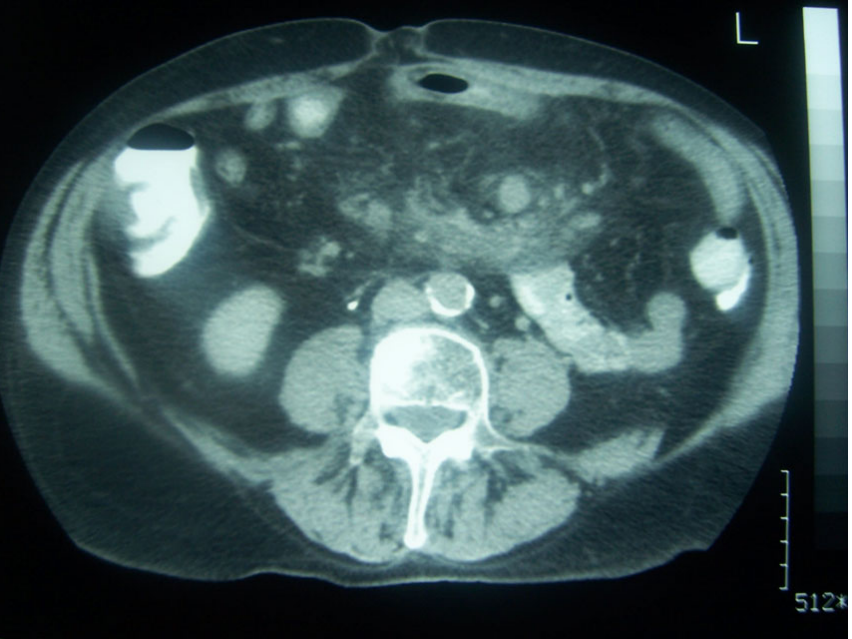
**CT scan of the abdomen showed a mass involving the mesentery at the edge of which lays a loop of small intestine with thickened wall**.

A thorough clinical examination including colonoscopy, gastroscopy, computed tomography (CT) scan and magnetic resonance imaging of the abdomen was performed. Endoscopic procedures revealed no pathology. On CT scan a mass was diagnosed involving the mesentery in close proximity with a loop of small intestine with thickened wall. (figure 2).

Laparotomy revealed a single 30 cm long stricture of the small intestine with brown discoloration extending approximately 10 cm and edema of the bowel wall and mesentery. Enlarged mesenteric lymph nodes were present as well.

Presuming Crohn's disease or other colitis, 80 cm length of small intestine was resected and anastomosed. Cholecystectomy was also performed due to cholelithiasis. The postoperative course of the patient was uneventful.

**Figure 3:**
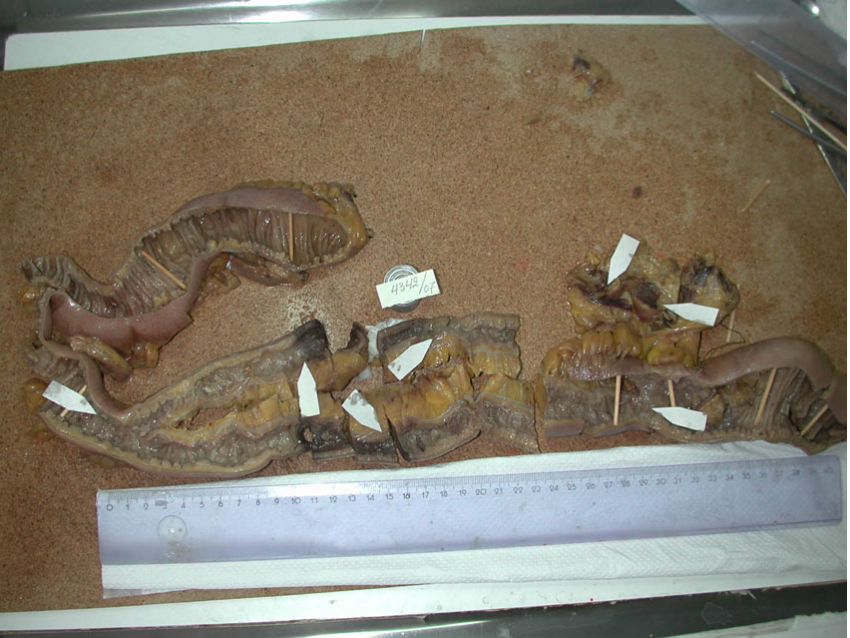
**Macroscopic view of the dissected small intestine**.

Grossly the specimen showed a stenosis of about 25 cm, the serosa in that region was thick with a bluish discoloration of 10 cm length. The mucosa was brown, friable with superficial ulcerations and absence of plicae mainly at the centre of the stenosis. The mesenterium was obvious thick with a nodular appearance (figure 3).

**Figure 4:**
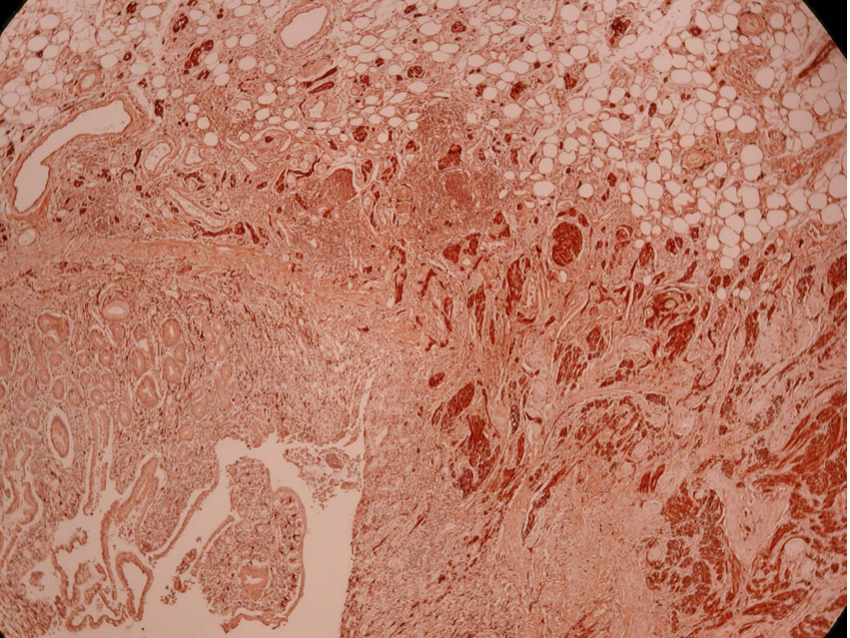
**Profound hyperplastic non-myelinated bundles of nerve fibres positive in S-100**.

**Figure 5:**
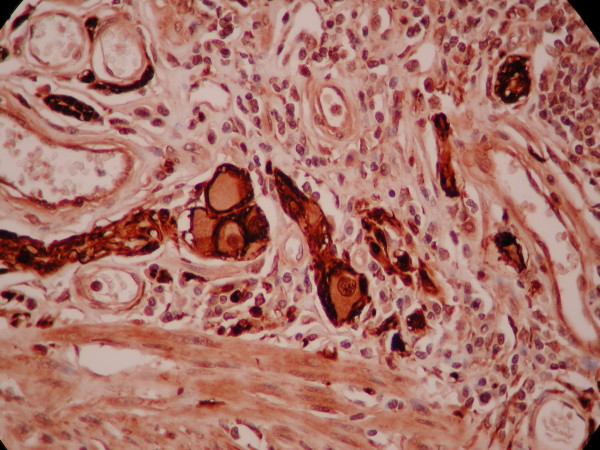
**Groups of abnormal ganglion cells S-100 positive**.

**Figure 6:**
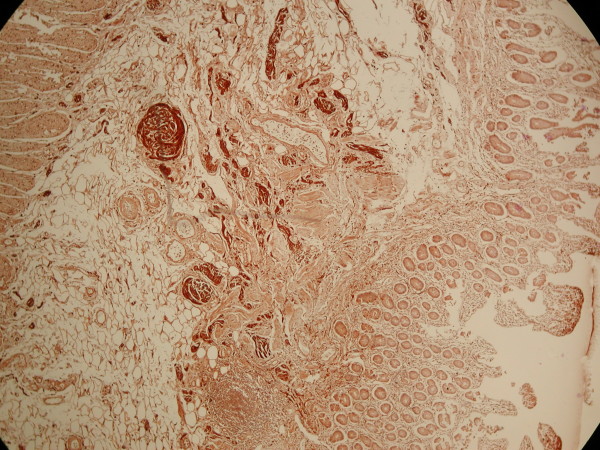
**Plenty of adipose tissue in submucosa with many haemangiomatous vessels and bundles of non-myelinated nerve fibres**.

Microscopically the submucosa was extremely thick with extensive fatty infiltration and mixing of abnormal intermingled non-myelinated nerve bundles, interspersed large ganglion cells, hyperplastic smooth muscle tissue that originates mainly from the thickened muscularis mucosa and less from the muscularis propria, and hemangiomatous vessels (figure 4, 5, 6).

The vessels showed muscular hypertrophy and locally ectasia and were observed also in the serosa. Small areas of fibrosis were seen everywhere. The mucosa was ulcerated and severe inflammed by lymphoplasma cells and less eosinophils. Foci of inflammation were seen in the subserosa. There was no transmural inflammation, granulomas or fissuring. No microorganism with histochemical stains was observed. Hyperaemic, thick wall vessels were observed in the mesenterium. The regional lymph nodes showed no significant morphological alterations.

## Discussion

The neuromuscular and vascular hamartoma of small bowel has been reported in <15 cases up to today [1-6]. In all these cases similar gross and morphological findings were found. In our case we noted also other characteristics. In gross examination there was only a long concentric stenosis measured 25 cm. Pathological features were the same as the previously described "NMVH", but a widely fatty submucosal infiltration was observed to the whole length of the stenotic region. These alterations were also described by Salas et. al. He proposed the term of "NMeH" in those cases with similar pathological characteristics of NMVH in which there is an additional mesenchymal tissue participation [1]. In our case a submucosal extensive infiltration of the adipose tissue was observed. The morphological features of NMVH can be observed in some other situations which cause luminal stenosis of the bowel such as thickening of muscularis mucosa. Shepherd et al. presented some cases where diagnosis of Crohn's disease or of NMVH was questionable [3]. Muscularization, hyperplasia of bundle nerves of the submucosa or hemangiomatous vessels can be also seen in Crohn's disease, ischemia, enteritis by radiation injury, etc. Therefore, the main differential diagnosis should be from Crohn's disease. In our case the hallmark features were absent: no transmural inflammation, no granulomas or fissuring of the mucosa were observed. The postoperative course of the patient was uncomplicated and now, six months later, he is free of any gut symptoms.

In summary, the gross characteristics and the morphological features, the clinical history and the postoperative health status of the patient support the diagnosis of Neuromesenchymal hamartoma of the small bowel.

## Consent

Written informed consent was obtained from the patient for publication of this case report and accompanying images. A copy of the written consent is available for review by the Editor-in-Chief of this journal.

## Competing interests

The authors declare that they have no competing interests.

## Authors' contributions

ET was responsible for editing, english editing, search of the literature. GV was responsible for english editing, correction, editorship of the manuscript. NS was responsible for the search of the literature. KP was responsible for english editing, correction. KM was responsible for the histology consulting and pathology examination. KC was responsible for editing.
